# A Novel Technique of Stenting of the Renal Artery In-Stent Restenosis with GuideLiner® through Radial Approach

**DOI:** 10.1155/2017/1742058

**Published:** 2017-06-04

**Authors:** Maheedhar Gedela, Shenjing Li, Tomasz Stys, Adam Stys

**Affiliations:** ^1^Department of Internal Medicine, University of South Dakota Sanford School of Medicine, Sioux Falls, SD, USA; ^2^Sanford Cardiovascular Institute, University of South Dakota Sanford School of Medicine, Sioux Falls, SD, USA

## Abstract

In-stent restenosis of the renal arteries is relatively common and its management is not well studied. An 83-year-old female with bilateral renal artery stenosis and balloon angioplasty and stenting bilaterally one year ago was found to have recurrent severe elevations in the blood pressure despite medical management. Renal artery duplex showed 60–99% stenosis of the right renal artery and 20–59% stenosis of the left renal artery. A subsequent angiography of the right renal artery revealed 80% in-stent restenosis at the ostium. We describe a new approach of balloon angioplasty and stenting through radial access site with the assistance of a GuideLiner in a complex in-stent restenosis of the renal artery.

## 1. Introduction

The estimated prevalence of renal artery stenosis (RAS) is more than 5% in people older than 50 years in the US [1]. Currently, there are no formal guidelines outlining the management of RAS for refractory hypertension. Although percutaneous transluminal renal artery angioplasty had good short-term outcomes, the restenosis rates were high, especially for ostial disease [2]. The stented vessel diameter of <5 mm and stents > 20 mm long increase the risk for restenosis [3, 4]. The available treatment options for recurrent RAS are percutaneous transluminal angioplasty and/or placement of a second stent, use of polytetrafluoroethylene- (PTFE-) covered stents, cutting balloon angioplasty, endovascular brachytherapy, excimer laser-assisted angioplasty, and surgical atherectomy [5, 6].

## 2. Case Presentation

An 83-year-old female with bilateral renal artery stenosis and balloon angioplasty and stenting bilaterally one year ago was found to have recurrent severe elevations in the blood pressure despite medical management. Her past history included hypertension and coronary artery bypass surgery. The laboratory evaluation revealed stable renal function with creatinine at 0.90 mg/dL (normal: 0.50–1.30). Renal artery duplex showed 60–99% stenosis of the right renal artery (RA) and 20–59% stenosis of the left renal artery. Renal arteries angiogram was performed.

## 3. Procedure Technique

**Figure 1:**
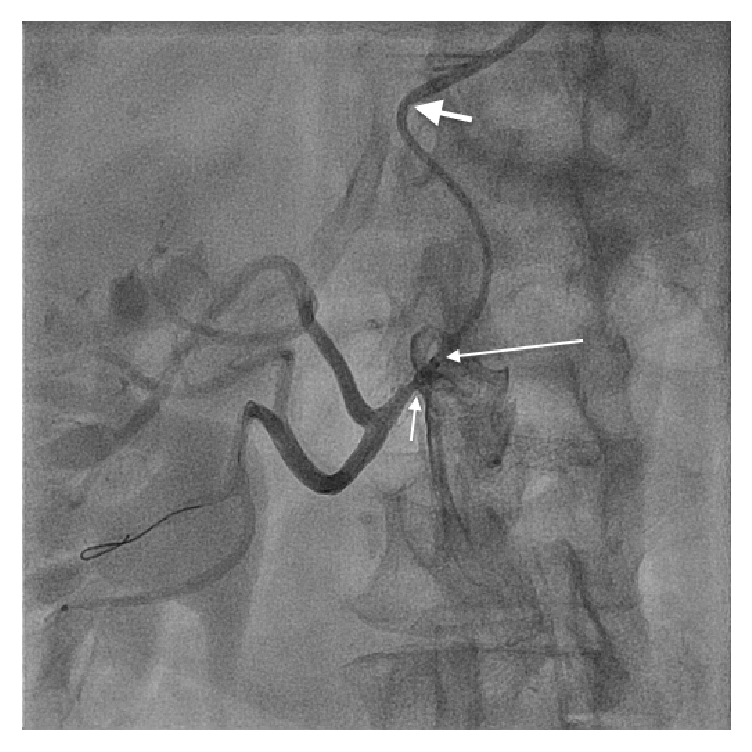
Renal artery ostial stenosis with distorted stent (short arrow). Long arrow represents tip of the GuideLiner and thick arrow represents tip of the Multipurpose guide catheter.

**Figure 2:**
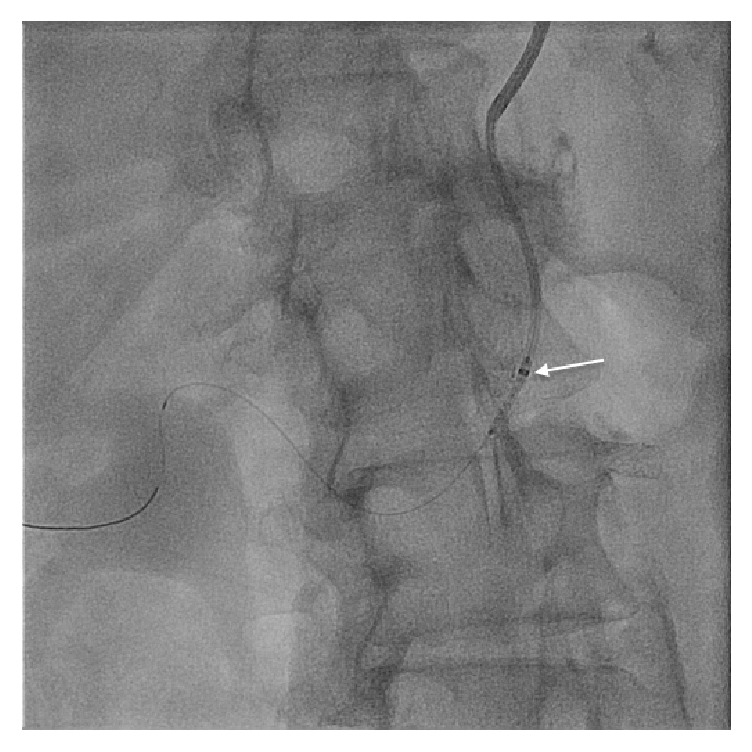
Delivery of the drug-eluting stent with the assistance of GuideLiner (arrow represents tip of the GuideLiner).

**Figure 3:**
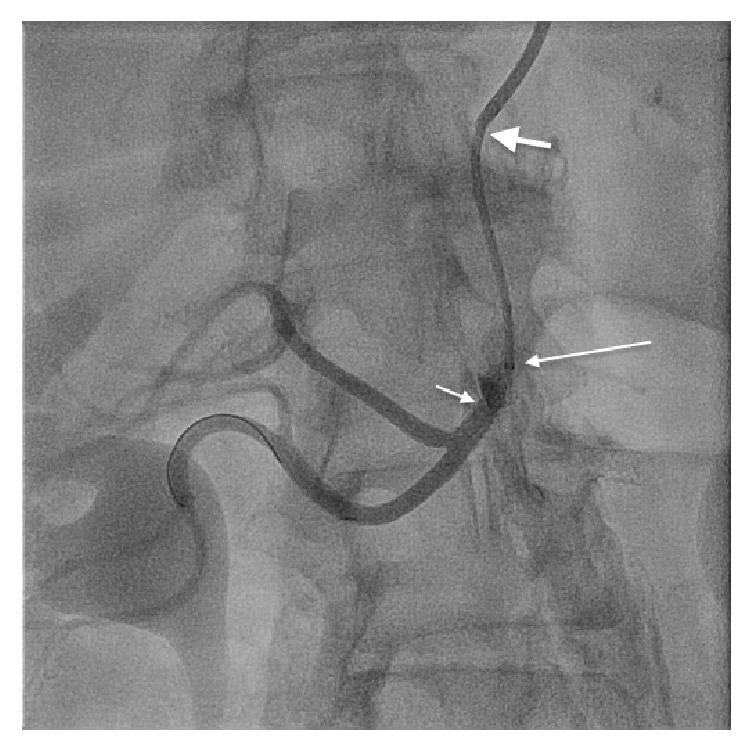
Good result after stenting (short arrow). Long arrow represents tip of the GuideLiner and thick arrow represents the tip of Multipurpose guide catheter.

A right radial access was obtained with a 5/6 French-Slender® sheath. A 5-French 125 cm JR4 diagnostic catheter was used for left renal angiography showing a mild mid in-stent restenosis of left renal artery. A Multipurpose 100 cm 5-French catheter was used for right renal angiography but was too short to reach the ostium. Thus, the 6-French 100 cm Multipurpose guide catheter was used and as it was also too short to reach the ostium, a 6-French GuideLiner (GL) extension was used to allow engagement of RA. Angiography of the right RA revealed 80% in-stent restenosis (ISR) at the ostium with hourglass like stent distortion at this site (Figure 1). The GL allowed endovascular intervention on right RA (Figure 2). A 190 cm BMW guidewire was used and stenosis predilated with a 4 × 15 mm balloon. A drug-eluting stent (DES) Xience 4 × 12 mm was deployed at high pressure covering the ostium of the right RA well. The stent was postdilated proximally and in mid-section with a noncompliant 4.5 × 15 mm balloon at high pressure. The procedure was performed with rapid exchange system. DES was used considering the relatively small renal artery size and ISR, with the hope of reducing restenosis. The result was very good angiographically (Figure 3). The patient tolerated the procedure well without any radial access site complications. At the 6-month follow-up clinic visit, the renal artery duplex showed 20–59% stenosis of the right RA and her blood pressure maintained less than 140/80 mmHg consistently.

## 4. Discussion

RAS is encountered in 6–18% of patients undergoing cardiac catheterization and 16–40% of patients undergoing arteriography for aortic and peripheral vascular disease [7]. A randomized controlled study showed 75% primary patency rate in combined angioplasty and stent group versus 29% in angioplasty group at 6 months in patients with ostial atherosclerotic RAS [8]. The true incidence of ISR is unknown due to the paucity of the prospective studies [2]. Based on observational studies, the reported rates of ISR following RA stent placement vary from 6 to 60%. Though early studies described the occurrence of ISR within the first 6–12 months, the subsequent analyses noted ISR occurrence anytime following RA stent placement [2, 4]. The recurrent ISR generally occurs from 4 months to 10 years and it is usually high [2]. In a small cohort study where the majority of interventions were performed through femoral access, the drug-eluting stenting showed 0% recurrence of ISR (*n* = 0/4) after a mean follow-up of 258 ± 163 days [4]. Another retrospective small study noted repeat stenting of recurrent ISR resulted in better patency rates compared to angioplasty alone [9]. Kakkar et al. reported wide patency of the renal artery at 6-month follow-up with paclitaxel-eluting stent implantation in a two-time ISR patient [10].

There is a paucity of data in the literature describing renal artery interventions through radial site access [11]. To the best of our knowledge, this is the first case illustrating the novel technique of percutaneous transluminal balloon angioplasty and DES placement with the assistance of GL through radial access in the complex ISR of the renal artery. Radial approach is beneficial for immediate sheath removal, prompt mobilization of the patients, low rate of access site complications, and a potential for utilization of fewer devices. In our case, the right RA take-off was favorable for radial access. However, radial access for renal artery interventions can be associated with technical difficulties of catheter advancement in the aorta, potential for distal embolization, and dependent on operator experience. In our case, the GL extension allowed reaching RA from radial approach, as guide catheter alone was too short for that. Additionally, we think it improved the precision of stent delivery.
